# SGLT2 Inhibitors in Chronic Kidney Disease: From Mechanisms to Clinical Practice

**DOI:** 10.3390/biomedicines10102458

**Published:** 2022-10-01

**Authors:** Roko Skrabic, Marko Kumric, Josip Vrdoljak, Doris Rusic, Ivna Skrabic, Marino Vilovic, Dinko Martinovic, Vid Duplancic, Tina Ticinovic Kurir, Josko Bozic

**Affiliations:** 1 Department of Nephrology, University Hospital of Split, 21000 Split, Croatia; 2 Department of Pathophsiology, University of Split School of Medicine, 21000 Split, Croatia; 3 Department of Pharmacy, University of Split School of Medicine, 21000 Split, Croatia; 4 Department of Pediatrics, University Hospital of Split, 21000 Split, Croatia; 5 Department of Endocrinology, Diabetes and Metabolic Diseases, University Hospital of Split, 21000 Split, Croatia

**Keywords:** SGLT2 inhibitor, chronic kidney disease, diabetes mellitus

## Abstract

In recent years, sodium-glucose co-transporter 2 inhibitors (SGLT2i) have demonstrated beneficial renoprotective effects, which culminated in the recent approval of their use for patients with chronic kidney disease (CKD), following a similar path to one they had already crossed due to their cardioprotective effects, meaning that SGLT2i represent a cornerstone of heart failure therapy. In the present review, we aimed to discuss the pathophysiological mechanisms operating in CKD that are targeted with SGLT2i, either directly or indirectly. Furthermore, we presented clinical evidence of SGLT2i in CKD with respect to the presence of diabetes mellitus. Despite initial safety concerns with regard to euglycemic diabetic ketoacidosis and transient decline in glomerular filtration rate, the accumulating clinical data are reassuring. In summary, although SGLT2i provide clinicians with an exciting new treatment option for patients with CKD, further research is needed to determine which subgroups of patients with CKD will benefit the most, and which the least, from this therapeutical option.

## 1. Introduction

Chronic kidney disease (CKD) of any etiology is on the rise on a global scale. Caused by multiple conditions, such as diabetic kidney disease (DKD), hypertension, glomerulonephritis and various infections, in 2017, it was estimated to affect ~10% of the world’s adult population [1], which represented a 29% increase since 1990 [2]. CKD has over time emerged as one of the leading causes of worldwide mortality [3]. The prevalence of type 2 diabetes mellitus (T2DM) is also rising rapidly worldwide, especially in the Western world, burdened by the concomitant rise in obesity [4]. T2DM is a complex endocrine and metabolic disorder with grave effects on multiple organ systems. One of these organ systems is the kidney, with DKD affecting up to a third of all diabetic patients and being one of the most feared chronic diabetic complications. Not only is DKD the most significant predictor of mortality for diabetic patients, but it is also the major cause of end stage renal disease (ESRD) [5].

SGLT2i received approval for treatment in T2DM nine years ago. In that period, they have evolved from a novel hypoglycemic agent, to a potent cardio- and renoprotective option in the T2DM and heart failure treatment arsenal, and recently, to an exciting standalone therapeutical prospect for CKD, regardless of the presence of T2DM. It all started with phlorizin, a natural product used to treat fever and infectious diseases, first isolated in 1835 by a French chemist [6,7]. About a century later, it was discovered that phlorizin, in fact, blocks glucose absorption in the kidney and small intestine by inhibiting the SGLT family of proteins [8]. SGLTs are a family of several members, in which the first two are found in the kidney. Phlorizin, however, is known to inhibit both of those, namely SGLT1 and SGLT2. While SGLT2 have been found solely in the kidney, SGLT1 has also been located in the small intestine, lung, heart and liver [9]. This suggested that SGLT1 has several extra-renal functions and that its inhibition would potentially lead to diverse side effects. Unfortunately, this, along with its low bioavailability, was a significant issue for phlorizin, as it was found to cause severe gastrointestinal effects, such as diarrhea and subsequent dehydration [7]. Nevertheless, a need for a selective SGLT2 inhibitor was born. 

This triggers the following question: what in fact makes the renal part of SGLT1/2 inhibition so attractive? Diving deeper into the mechanism, the glucose reabsorption in the kidney is facilitated by active sodium removal by the Na+/K+-ATPase on the basolateral membrane, which creates the electrochemical force for glucose entry via sodium driven sodium-glucose cotransport with the SGLT1/2 through the apical membrane [10,11]. Glucose then re-enters the bloodstream primarily via GLUT2 on the basolateral side [12]. In physiological conditions, the renal proximal tubule reabsorbs all of the filtered glucose (~180 g/day) [10,13,14]. Of that, SGLT1, situated in the segment 3 of the proximal tubule, is responsible for 3–10% of the filtered glucose, while the rest is reabsorbed by SGLT2 in segments 1 and 2 [15,16,17,18,19]. In healthy kidney individuals, the kidney filters 160–180 g of glucose daily, while the renal transport maximum of glucose is reached around 300–500 mg/min [20]. The surplus glucose is excreted with urine, which can then lead to polyuria and polydipsia, common symptoms of diabetes. This mechanism acts as a ‘safety valve’ that prevents extreme hyperglycemia. SGLT2 inhibition, however, leads to a decline in the renal absorptive capacity for glucose to ~80 g/day. Consequently, the valve opens at a lower threshold, and subsequently more glucose is excreted with urine [21]. Therefore, it is clear why SGLT2 inhibitors were deemed a potent anti-hyperglycemic prospect and significant time and effort was put into their development. In 2013, the first SGLT2 inhibitors, dapagliflozin and canagliflozin, were approved for use. In the meantime, ipragliflozin, empagliflozin and ertugliflozin were added to the group. On a separate note, even though the development of selective SGLT2 inhibitors (SGLT2i) has been a priority for reasons previously explained, it is worth mentioning that a dual SGLT1 and SGLT2 inhibitor by the name of sotagliflozin has, in recent years, been approved for the treatment of both T1DM and T2DM [22]. 

After almost a decade since the approval of the first inhibitor, the SGLT2i now represent a mainstay in the therapeutical guidelines of T2DM and HF. What is most interesting, and is in fact the focus of this article, is that SGLT2i have been widely shown to also provide substantial renoprotective effects, thus further expanding the plausible implications of this drug [23]. Some authors even argue that the benefits of these medications raise the question of whether SGLT2i should also be used as primary prevention in healthy individuals [24]. On the other hand, others warn that due to “worldwide inertia“, SGLT2i are commonly not prescribed in situations where they would be appropriate, such as patients with cardiorenal risks [25]. Hence, in the present study, we aimed to discuss the pathophysiological target points by which SGLT2i may offer benefits in CKD, and to summarize the available clinical studies that explored the effects of this drug in the above-noted population.

## 2. Renoprotective Effects of SGLT2 Inhibitors

### 2.1. Direct Effects

**Figure 1:**
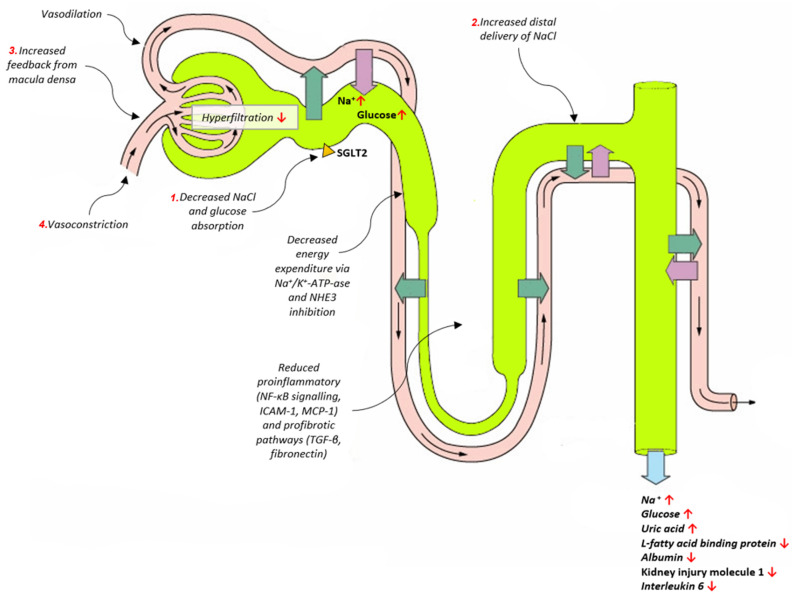
Direct mechanisms by which SGLT2 inhibitors exert renoprotective effects. Multiple mechanisms conjoin and result in normalization of glomerular filtration. Green arrows represent reabsorption from kidney tubules, purple arrows indicate secretion, whereas blue arrow indicates excretion. As indicated by numbers 1–4, inhibition of SGLT2 prevents renal sodium and glucose reabsorption, thus increasing sodium delivery to macula densa, which in turn restores tubuloglomerular feedback by causing afferent arteriolar vasoconstriction through increased adenosine production and intracellular calcium levels. Abbreviations: SGLT2: sodium-glucose co-transporter 2; NHE3: the Na+-H+ exchanger 3; NF-κB: nuclear factor kappa-light-chain-enhancer of activated B cells; ICAM-1: intercellular adhesion Molecule 1; MCP-1: monocyte chemoattractant protein-1; TGF-β: transforming growth factor β; ↑: increase in; ↓; decrease in.

With the mechanism of SGLT2i that occurs in the renal proximal tubule, the effects on the kidney itself, both direct and indirect, are unsurprisingly numerous (Figure 1).

Firstly, SGLT2 inhibition reduces glomerular hyperfiltration, a pathophysiological mechanism that operates in T2DM, leading to the progression of CKD [26,27,28]. In hyperglycemia, the reabsorption of sodium and glucose in the proximal tubule is greatly increased, which decreases the delivery of sodium to macula densa [18,29]. The tubule-glomerular feedback (TGF) is then decreased, the afferent arteriole dilates and the renal blood perfusion and vascular wall pressure increases, which then leads to glomerular hyperfiltration, and in the long term, injury [18,29,30,31]. By inhibiting the SGLT2, the sodium and glucose reabsorption is decreased, the tubule-glomerular feedback is restored to normal, and the aforementioned damage is undone. Another significant factor is the association of SGLT2 and the Na+-H+ exchanger 3 (NHE3). Several studies have reported SGLT2i-induced suppression of NHE3, which further explains its natriuretic effect [32,33,34]. These mechanisms also shed light on a slight fall in the estimated glomerular filtration rate (eGFR) that occurs upon commencing the SGLT2i therapy, an effect that, at first, cast doubt on the safety of SGLT2i, but has since been shown to be transient and with no short- or long-term negative consequences [35,36,37]. This fall is a sort of an antithesis to the eGFR rise, or the so-called hyperfiltration that comes with prolonged hyperglycemia, and is, in fact, widely argued to be renoprotective in the long term [38]. Interestingly, van Bommel et al. have shown that dapagliflozin treatment decreases filtration fraction without increasing renal vascular resistance, suggesting that SGLT2 inhibition’s reducing effect on glomerular filtration is due to post-glomerular vasodilation, rather than pre-glomerular vasoconstriction [39]. That makes this well-known SGLT2i effect, at least partially, TGF and glucose-independent [40], thus providing a viable explanation for the renoprotective effects irrespective of the presence of DM.

Secondly, in order to fulfill its metabolic demand, the kidneys use up an unproportionally large amount of energy, as much as 10–15% of glucose [41]. As early as 1994, Körner et al. showed that phlorizin treatment of streptozocin-induced diabetes rats reduced cortical and total renal oxygen consumption as a direct consequence of reduced Na+/K+-ATPase activity [42]. The very absence of, or merely a reduction in, this activity provided by the SGLT2 inhibition protects the organ from hypoxia, which is a common pathway to progression of CKD by promoting and sustaining fibrotic and inflammatory response [43,44]. An animal study that studied streptozocin-induced diabetic rats measured renal O_2_ homeostasis, verifying that diabetic kidneys displayed reduced baseline pO_2_ in both cortex and medulla, while SGLT inhibition by phlorizin improved cortical pO_2_, but reduced medullary pO_2_. The latter finding, interestingly, suggests a possible redistribution of active transport to distal nephron segments, resulting in the observed medullary hypoxia [45]. Another animal study treated hypoxic human kidney 2 (HK2) cells with dapagliflozin, reaching a conclusion that dapagliflozin reduces renal damage by inducing renal expression of nephroprotective hypoxia-inducible factor 1 (HIF1) and associated proteins [46]. 

An in vitro study by Ishibashi et al. further explained how SGLT2 inhibition ameliorates hypoxia, proposing that SGLT2-mediated glucose entry into the tubular cells is central to oxidative stress and inflammatory response that occurs in proximal tubular cells [47]. An early study by Vallon et al. concluded that SGLT2 inhibition is “not critical“ for the induction of renal growth and markers of renal injury, inflammation and fibrosis [48]. However, further worldwide research has found the exact opposite, including a follow up study by the aforementioned group of authors [40]. In fact, SGLT2i have since been consistently found to decrease albuminuria and urinary excretion of tubular injury and inflammatory markers, such as L-fatty acid binding protein [49], kidney injury molecule 1 (KIM-1) and interleukin 6 (IL-6) [50,51], where the former effect is most likely a result of the previously discussed decrease in intraglomerular pressure, and therefore not necessarily tied to the glucose lowering effect of SGLT2 inhibition. In vitro studies in human proximal tubular cells have also found that SGLT2 inhibition induced a reduction in the plasma levels of a number of molecules related to inflammation, extracellular matrix turnover and fibrosis, such as TNF receptor 1 (TNFR1), matrix metalloproteinase 7 (MMP7), IL-6 and fibronectin 1 (FN1) [51,52,53,54]. Similarly, Ojima et al.’s study on diabetic rats proposed that SGLT2 inhibition reduces oxidative, inflammatory and fibrotic responses in the kidney, partly via suppression of the advanced glycation endproducts and the receptor for advanced glycation endproducts (AGE-RAGE) axis, which is an oxidative stress-induced proapoptotic pathway [49]. In addition, SGLT2i were shown to reduce glomerular mesangial expansion, macrophage infiltration and interstitial fibrosis in a dose-dependent manner [55]. A vast majority of these studies were carried out in the presence of diabetes, but the discussed evidence suggests effects that go beyond diabetes, while its presence might merely amplify them. A significant reason as to why those effects are markedly amplified in DM is the fact that SGLT2 expression is upregulated in this population, as shown in both animal and human studies [27,56,57,58]. This is also the case for the previously discussed effects on TGF and renal oxygen consumption. 

To summarize the three broadly divided mechanisms, the first one, the reduction in hyperfiltration, and therefore glomerular injury, is DM-related through the TGF, but has also been speculated to be glucose-independent. The second, the reduction in energy consumption, and therefore hypoxia, is by definition glucose-dependent and greatly amplified in the presence of diabetes, although in theory, it occurs even in the absence of it. The third, the inhibition of inflammatory, fibrotic and proapoptotic response, to a significant extent does not necessarily relate to diabetes. This shows the great scope of SGLT2 inhibition’s direct effects on the kidney and provides explanations for its effectiveness in both diabetic and non-diabetic chronic kidney disease.

### 2.2. Indirect Effects

Having discussed the direct effects of SGLT2 inhibition on the kidney at length, in this subsection, we will acknowledge the many indirect effects of SGLT2 inhibition on the kidney, whether this is through changes in hormones, metabolism or reduction in other risk factors for CKD. 

SGLT2 inhibition-induced glycosuria improves endogenous glucose production, pancreatic beta-cell function and insulin sensitivity. Consequently, it reduces insulin and increases glucagon secretion, shifting substrate utilization from carbohydrate to lipid [58,59,60,61,62]. Those effects are beneficial for the kidney, since insulin promotes proliferation of the extracellular matrix, which damages renal function, whereas glucagon maintains renal function by dilating blood vessels, increasing renal filtration and electrolyte excretion [51,58,59,60,61,62,63].

Multiple studies have reported other glucosuria-related effects of SGLT2i on multiple conditions that can have a profound impact on the kidneys, such as body weight and blood pressure. For example, obesity is known to increase renal blood flow and glomerular filtration, leading to albuminuria and long-term injury [64,65]. A great number of studies have shown a significant beneficial effect of SGLT2i on obesity and body weight, an effect that has been strongly linked with glucosuric effects of SGLT2 inhibition, but also improved insulin resistance and glucose tolerance [51,61,66,67,68,69]. The effect has been proven even in patients without diabetes mellitus in a study by Bays et al., who found significant reductions in body weight after daily administrations of canagliflozin in a 12-week period [70]. Subsequent studies have confirmed this finding, associating renal glucosuria independent of diabetes with lower body weight and also lower blood pressure [66]. 

With regard to blood pressure, multiple studies and meta-analyses have found a beneficial effect of SGLT2 inhibition on hypertension [68,69,71,72,73]. Hypertension has long been known to exert damage on renal vessels, and thus have vast negative consequences on renal function. Even though the primary mechanism of blood pressure reduction in SGLT2 inhibition is believed to be the plasma volume reduction that comes with the natriuresis, a number of studies link the hypotensive effect of SGLT2i to sympathoinhibition, demonstrating SGLT2 inhibition induced reduction in tyrosine hydroxylase and norepinephrine elevations [73,74], and detecting no rise in heart rate, despite the observed reductions in blood pressure and plasma volume, suggesting a dampening of sympathetic nervous system activity [72]. 

Although dyslipidemia does not seem to have a direct impact on the progression of CKD, its detrimental effect on the kidney is funneled through damage to the cardiovascular system, which is, in fact, in many ways associated with CKD. Dyslipidemia and CKD, thus, form a sort of a *circulus vitiosus*, as CKD is known to decrease high-density lipoproteins (HDL) and increase triglycerides, while nephrotic syndrome is even more atherogenic by increasing low-density lipoproteins (LDL) and total cholesterol (TC) [27]. SGLT2 inhibition has been shown to ambiguously affect dyslipidemia, as shown both in animal and human studies. SGLT2i seem to increase the levels of LDL via reduced clearance from circulation, while reducing levels of plasma triglycerides, and having no effect on HDL [51,75,76].

SGLT2i have many protective effects on endothelial cells, such as promoting proliferation, migration and differentiation of said cells, while also increasing the bioavailability of nitric oxide derived from endothelium, which subsequently promotes vasodilation in multiple organs, including the kidneys itself, thus reducing the burden on the organ [73,77]. Hyperuricemia is another detrimental factor for endothelial cells, but it also mediates inflammatory response and forms deposits in the kidney, which can cause damage to the tubules. SGLT2i have a decreasing effect on uric acid levels, as shown by two meta-analyses, of which the more recent one by Yip et al. in 2022 determined a beneficial effect of SGLT2 inhibition on reducing serum urate concentrations in patients with and without DM [78]. In 2018, however, Zhao et al. found that this effect was abolished in patients with eGFR < 60 mL/min per 1.73 m^2^ [79]. The putative mechanism of the uric acid lowering effect by SGLT2i is thought to be mediated by the efflux of uric acid through GLUT9 in the proximal tubule. The absence of the uric acid lowering effect in CKD is partially thought to be mediated by the lack of GLUT9 efflux, but for the most part, owing to the fact that reduced filtration of uric acid in these patients overcomes the reduction in reabsorption mediated by SGLT2i.

Hematocrit and the reticulocyte levels increase with SGLT2i, an effect that was assumed to be a consequence of the diuretic effect. However, it is theorized that this occurs also because of the enhancement of erythropoiesis, which is a result of an increase in erythropoietin levels (80). Taken together, the increase in hematocrit during SGLT2 inhibitor therapy may indicate improvement of hypoxia and oxidative stress in the tubulointerstitial region of the renal cortex, as well as recovery of erythropoietin production by “neural crest-derived” fibroblasts [80,81].

As previously mentioned, SGLT2i have profound benefits on the cardiovascular system. SGLT2i have been observed to significantly lower the risk of not only renal, but heart failure as well [23,82,83,84,85,86,87,88,89], which is not surprising considering the many effects already discussed in this chapter, such as the improvement of blood pressure, reduction in obesity and promotion of vasodilation, all of which have a beneficial effect on ventricular preload and afterload [84]. However, the most prominent effects of SGLT2i on heart function are independent of the aforementioned cardiovascular risk factors, which is in fact why they were endorsed in the contemporary heart failure guidelines as Class I recommended therapy [90]. Nevertheless, the mechanisms by which SGLT2i constitute favorable effects on populations with heart failure are beyond the scope of the present review. SGLT2i also have a significant association with lower risks for vascular diseases, including atherosclerosis, hypertension, hypertensive emergency and varicose vein [84]. This finding is speculated to be a direct consequence of the above-mentioned and previously discussed mechanism of SGLT2i; the increased bioavailability of endothelium-derived nitric oxide, which inhibits the contraction of vascular smooth muscle cells [84]. A recent meta-analysis has even reported a lower risk of bradycardia and atrial fibrillation, suggesting the possible antiarrhythmic efficacy of SGLT2i [84], which could be an indirect consequence of all the benefits on the heart put together, as well as better electrolyte regulation that has been associated with SGLT2i. Furthermore, SGLT2i are reported to reduce the risk of cardiovascular mortality [88,89], which is a major factor in CKD patients’ mortality.

## 3. SGLT2 Inhibitors in CKD—Clinical Evidence

### 3.1. Diabetic Kidney Disease

Over the last decade, there has been a great number of studies that have analyzed the clinical outcomes of SGLT2i on the kidney. A vast majority of them studied patients with DKD. Clinical evidence is summarized in Table 1. 

The EMPA-REG OUTCOME trial studied 6952 patients with DKD categorized into subgroups of albuminuria and eGFR, reaching a conclusion that empagliflozin significantly reduced kidney outcomes and reduced yearly loss of eGFR, which was consistent across all subgroups [91]. A meta-analysis in 2019 obtained data from 27 studies and 7363 participants with T2DM and CKD of eGFR < 60 mL/min/1.73 m^2^. It found that SGLT2i attenuated the annual decline in eGFR and reduced the risk of composite renal outcomes [92]. Another meta-analysis in 2019 obtained data from 25 studies and 43,721 participants with T2DM, with and without CKD. The conclusion was that SGLT2i slowed eGFR decline, lowered albuminuria progression, improved adverse renal endpoints and reduced all-cause mortality [93]. An exploratory analysis of the EMPA-REG OUTCOME trial confirmed both short and long-term benefits of empagliflozin on ACR in patients with T2DM, irrespective of albuminuria status at baseline [94]. An additional analysis of the EMPA-REG OUTCOME trial concluded that empagliflozin reduces the risk “incident or worsening nephropathy“, while patients who were using angiotensin-converting enzyme inhibitors (ACEi) or angiotensin receptor blockers (ARBs) had a higher risk of acute renal failure, although it was reduced in groups that were given empagliflozin [95]. A similar conclusion has been drawn for dapagliflozin, with three smaller studies on patients with T2DM and RAAS blockade therapy, all finding a reduction in albuminuria by 31–42% [96,97,98]. Similarly, a large study on 7020 patients with T2DM, out of which 84% were taking renin-angiotensin-aldosterone system (RAAS) blockade therapy, found that dapagliflozin improved clinical outcomes and reduced mortality consistently across the categories of eGFR and albuminuria [99]. As previously discussed, there have been plenty of concerning reports of a transient initial fall in eGFR of 3–5 mL/min/1.73 m^2^ in patients started on SGLT2i, seemingly with no effect on the later outcomes [35]. A study analyzed data from the EMPA-REG OUTCOME trial and found the ‘eGFR dip’ was more likely to occur in patients with a higher KDIGO (kidney disease improving global outcomes) risk category and diuretic therapy, but confirmed that the effect was transient and did not have an impact on renal outcomes [36]. A later analysis of the CREDENCE trial had the same conclusion [37]. Furthermore, a meta-analysis that included four studies on three different SGLT2i—EMPA-REG OUTCOME, CANVAS, CREDENCE and DECLARE-TIMI 58—with a total of 38,712 participants with T2DM concluded that SGLT2i reduced the risk of dialysis, transplantation, death due to kidney disease and provided protection against acute kidney injury [100].

Comparing dapagliflozin to a placebo over 6 weeks in patients with T2DM, a small randomized double-blind study found that dapagliflozin decreased albuminuria by 43.9% and eGFR by 5% [101]. It also decreased urinary excretion of KIM-1 and IL-6, suggesting that SGLT2 inhibitors ameliorate renal inflammation and ischemic proximal tubular injury, as previously discussed. In an effort to understand how SGLT2 inhibitors affect albuminuria, the same study found a lack of effect on the IgG-to-IgG4 and IgG-to-albumin clearance ratio, which suggests that SGLT2i do not alter the charge or size selectivity of the glomerular filtration barrier; hence, their beneficial effect on albuminuria, the study concluded, can only be explained by the reduction in intraglomerular pressure and/or improved tubular reabsorption [101].

A meta-analysis showed that a reduction in albuminuria with dapagliflozin cannot be predicted by baseline characteristics or changes in most of the other metrics, such as HbA1c and body weight. Urinary albumin to creatinine ratio (UACR) reduction, although proven with SGLT2i, remains an individual characteristic with 46% patients in the study classified as ‘‘non-responders’’ [102]. 

Many studies analyzed both the renal and cardiovascular outcomes of SGLT2 inhibition therapy, reaching various interesting conclusions related to different sets of patients, according to the presence of different cardiovascular morbidities. An analysis of the CANVAS program, which studied the effects of canagliflozin versus a placebo on 10,142 patients with T2DM and eGFR > 30 mL/min/1.73 m^2^, concluded that canagliflozin improves renal outcomes independent of the baseline level of kidney function and a history or high risk of cardiovascular disease [103,104]. Similarly, in a randomized controlled trial with 4124 patients with high cardiovascular risk, empagliflozin was associated with slower progression of kidney disease and lower rates of clinically relevant renal events, such as eGFR reduction and initiation of RRT [105]. In patients with heart failure and T2DM, empagliflozin has been found to slow the rate of eGFR decline and lower the risk of serious renal outcomes, as shown by a randomized controlled study that included 3730 patients with EF < 40% [106]. A meta-analysis in 2019 included data from 3 trials and 34,322 patients with T2DM, where 60.2% had an established atherosclerotic cardiovascular disease. It found that SGLT2i have, apart from moderate benefits on major adverse cardiovascular events, ‘‘robust benefits’’ on the progression of renal disease, regardless of existing atherosclerotic cardiovascular disease or a history of heart failure [107].

In a randomized controlled trial that included 4401 patients with T2DM and albuminuric CKD, canagliflozin reduced the risk of end-stage renal kidney disease, a doubling of creatinine level, or death from renal causes by 34% [108]. Significantly, as SGLT2i have predominantly been contraindicated in patients with very low rates of eGFR, a subgroup analysis of the CREDENCE trial found that canagliflozin effects on renal outcomes are consistent even in patients with eGFR < 30 mL/min [109].

As shown, the studies about SGLT2i usage in DKD patients are numerous and the clinical evidence for their benefit is vast. The same cannot yet be said for the population of patients without DM; however, the existing evidence is worth a closer look.

### 3.2. CKD in Patients without DM

Heerspink et al. have been one of the first and most consistent groups in discussing the possibility of SGLT2i having beneficial effects on the kidney, independently of diabetes. In line with this, one of their early studies in 2016 showed that canagliflozin slows the progression of CKD, independently of its glycemic effects [110]. However, this was only the beginning [111]. While their renoprotective effect has long been suspected to be independent from the presence of diabetes, studies that support this hypothesis began to emerge in 2020. However, not all agreed. DIAMOND was a randomized, double-blind, placebo-controlled crossover trial that included 57 adult patients with CKD and without DM. The study showed no effect of dapagliflozin on proteinuria, whilst inducing an acute and reversible decline in eGFR. One positive outcome, however, was a reduction in bodyweight [112]. Shortly after, that conclusion was eclipsed by a meta-analysis of DAPA-HF and EMPEROR-Reduced trials, which combined 8474 patients suffering from heart failure with reduced ejection fraction (HFrEF), with or without DM and reached a conclusion that both dapagliflozin and empagliflozin improved renal outcomes for both sets of patients [113,114]. Perhaps the most important study was the DAPA-CKD trial with 4304 participants with GFR of 25–75 mL/min/1.73 m^2^ and ACT of 200–5000 mg/g. The DAPA-CKD concluded that regardless of the presence or absence of diabetes, the risk of a composite of a sustained decline in the estimated GFR of at least 50%, end-stage kidney disease, or death from renal or cardiovascular causes was significantly lower with dapagliflozin than with the placebo [115]. The scientific community now awaits the results of the EMPA-KIDNEY trial, which was recently reported to be “stopped early due to clear positive efficacy in people with chronic kidney disease“ [116].

### 3.3. SGLT2i across Different Populations

Having experienced this success in the adult population, SGLT2 inhibitors are thought to have the same effect in children; however, their use has not yet been approved due to a lack of data. Nevertheless, it is believed that “no pathophysiological clues exclude their application” [117] in children, which goes in line with the conclusions of this review. At the time of writing, canagliflozin and empagliflozin are undergoing phase 3 study in children and adolescents [118,119], while dapagliflozin has already been shown to be safe in pediatric populations by a phase 3 study [120]. The aforementioned studies predominantly include children with diabetes; however, taking everything into consideration, there seem to be no apparent reason why SGLT2i would not show the same efficacy and safety in children with CKD and no DM as they have in adults.

Relevant studies have found similar effects in regard to sex [121,122] and race [123]; however, a study last year has shown that black race, female sex and low socioeconomic status are associated with a lower use of SGLT2i [124], which is, therefore, a significant area of potential improvement.

Another important population that currently lacks evidence for the use of SGLT2i is the kidney transplant patients, since most large studies automatically do not include them due to safety concerns. The existing data, although limited, do show a modest impact of SGLT2i on glycemic control, body weight, serum uric acid levels and blood pressure, while “the frequency of reported adverse effects does not appear to exceed those found in nontransplant patients” [125]. Further research here is warranted.

All in all, the SGLT2i are slowly but surely being proven as effective and safe in all relevant populations, with no concerning evidence being forwarded as of yet.

### 3.4. Current Clinical Recommendations for SGLT2i Use in CKD

SGLT2i are a cornerstone of clinical guidelines and recommendations for treatment of HF, DM and now, as is the focus of this review, CKD as well. This is due to the previously discussed studies that have propelled them into worldwide use, SGLT2i recommendations, such as the United Kingdom Kidney Association (UKKA) guidelines, are subsequently incorporated in patient settings that were included in the aforementioned studies. Therefore, the eGFR and UACR cut offs for SGLT2i use in both diabetic and non-diabetic kidney disease are set to ≥25 mL/min per 1.73 m^2^ and ≥25 mg/mmol, respectively. Interestingly, the UKKA guidelines recommend initiation of SGLT2i in patients with eGFR 25–60 mL/min per 1.73 m^2^ irrespective of DM, and even in the case of albuminuria when there is a need to modify cardiovascular risk, thereby considering the powerful cardioprotective effects of SGLT2 inhibition [126]. It is notable that SGLT2i are, apart from the previously discussed UKKA guidelines, currently widely recommended only to albuminuric CKD patients. However, as the recent EMPA-KIDNEY trial included adults with CKD regardless of albuminuria, and since it has been stopped early due to overwhelming efficacy, it is likely that the updated recommendations will expand the population eligible for therapy with SGLT2i to non-albuminuric patients as well [127].

For similar reasons, with regard to the patients studied in the most important trials, such as CREDENCE and DAPA-CKD, SGLT2i are currently, as previously mentioned, not recommended to kidney transplant patients, and likewise, to patients that have taken any immunological therapy in the prior 6 months. Apart from DKD, other etiologies of CKD that have been studied in large trials, and therefore included in recommendations, are as follows: ischemic nephropathy, IgA nephropathy, FSGS, chronic pyelonephritis and chronic interstitial nephritis [126,127].

Whereas in DM settings, SGLT2 inhibitors are usually combined with metformin [128], in non-diabetic CKD, it is recommended to combine SGLT2 inhibition with RAAS blockade, wherever it is indicated and tolerated [126]. Having been up until the recent discoveries regarding SGLT2 inhibitors the only medications known to attenuate progression of CKD, the RAAS blockers were a noted part of many, if not most, of the discussed trials about SGLT2i, as shown in Table 1. Hence, their combination is not only proven to be safe, but also likely to have a synergistic effect in battling CKD progression.

Due to their pharmacokinetics, all SGLT2i are taken once daily. Being the medication with the most clinical evidence for non-diabetic CKD, 10 mg of dapagliflozin is currently the most frequently used SGLT2i [126] in this setting, but it should soon be joined by empagliflozin of 10 or 25 mg due to the incoming EMPA-KIDNEY trial. Canagliflozin and ertugliflozin are currently given in DKD in dosages of 100–300 mg and 5–15 mg, respectively [126].

In conclusion, SGLT2i have already become an important part of our therapeutical arsenal versus DM, DKD and non-diabetic CKD. The latter setting is dominated by the most frequently studied dapagliflozin, but at this rate, it will soon be enhanced by the arrivals of the others. There is a similar tendency of widening the scope of indications and reducing the number of counterindications due to the unknown, which will, hopefully, and most significantly, result in the addition of non-albuminuric CKD to worldwide recommendations, and also the inclusion of the currently excluded kidney transplant patients.

## 4. Adverse Effects

There have been various studies that have assessed the potential adverse effects of SGLT2i. One of the more discussed potential adverse effects of SGLT2i is the apparent risk of urinary tract infections (UTIs) and genital infections. A meta-analysis carried out in 2017 verified an increased risk for the latter, but not the former [129]. This view has been confirmed by the following studies in 2019 [23,130,131,132]; however, one reported that dapagliflozin does increase the risk of UTIs, although the exact mechanism was unclear [132]. Apart from rare studies, such as a recent one from Borovac et al., which did not find an increased risk of UTIs in a population with heart failure [133], unfortunately, the most recent meta-analyses paint a different picture, showing an increased risk for UTIs [82,134] that is consistent across all SGLT2 inhibitors [135], while reaffirming the previously established increased risk for genital and fungal infections [71,82,134,135,136]. While the probable cause for this—the greater flow of glucose through the urinary tract and the genital area—is abundantly clear, the opposite mechanism is probably what explains this interesting finding from a recent study [82]; SGLT2i are reported to lower the risk for pneumonia, bronchitis and respiratory tract infection, which is potentially caused by their general glucose lowering effect. This, however, is unlikely to explain why SGLT2i are, in the same study, associated with lower risks of asthma, chronic obstructive pulmonary disease and sleep apnea syndrome. Further research and possible confirmation of these findings are warranted.

In the previous years, several case reports and observational studies associated SGLT2i with acute kidney injury (AKI), presumably through the mechanism of increased diuresis and subsequent volume depletion. Nevertheless, all the relevant studies in the meantime have not only heavily rejected that notion [132], but have also shown that SGLT2i greatly decrease the risk for AKI [23,135,136,137,138].

For many years, there has been a concern that SGLT2i might be associated with euglycemic diabetic ketoacidosis (DKA). A possible mechanism was found in their insulin-independent reduction in blood glucose and ensuing hyperglucagonemia [139]. Similar to the previously discussed UTIs, the earlier studies rejected the apparent increased risk for DKA [132], while the most recent ones heavily reaffirm the risk [82,135,136,140].

Considering their principal mechanism of action, i.e., reducing blood glucose, SGLT2i have been suspected to cause hypoglycemia. However, studies have shown that the risk is increased only when SGLT2i are combined with other glucose lowering agents, such as insulin or sulfonylureas [23], which casts doubt on whether SGLT2i have a role in causing hypoglycemia and/or if it is a problem of mismanaged therapeutic combination. The latter theory is reinforced with new studies that report a ‘‘reduced trend’’ in the risk of severe hypoglycemia [135], which might be due to clinicians’ reaction to the combined risk of glucose lowering agents. Further research that focuses on the risk of hypoglycemia with combined diabetic therapy including SGLT2i might be needed, with a look into potential modifications of the therapeutic guidelines regarding the putative risk.

Similar to the previously discussed hypoglycemia, SGLT2i have also been hypothesized to cause hypotension, since one of the main consequences of increased glucose and sodium reabsorption in the proximal tubule is increased diuresis and possible volume depletion. All the recent studies have, to a lesser or greater extent, confirmed that hypothesis, putting the focus on the need to adjust diuretic and/or antihypertensive treatment accordingly [23,71,82,129,135,136].

Perhaps the most controversial topic regarding SGLT2i adverse effects has been their effect on bone metabolism and fractures. It is believed that increased diuresis induced by SGLT2i might disturb calcium and phosphate homeostasis, leading to bone mineral losses [129]. However, currently, there is no consensus, with some studies rejecting the increased risk of fractures for all SGLT2i [88,132,141], others claiming an increased risk for some of them and not the others [23,142], and some reporting an “increased trend“ in fractures across all four SGLT2i [135].

SGLT2i have also been associated with a higher risk of lower limb amputation, notably canagliflozin [143], while no such risk was associated with the use of empagliflozin [144]. Further studies rated the risk of amputation as very low [23,132,145], while only one meta-analysis [135] showed increased risk across all SGLT2i. Interestingly, a recent study [146] has associated reductions in body weight and blood pressure to lower limb complications and peripheral arterial disease in patients taking canagliflozin, which shows that SGLT2i-induced volume depletion might contribute to circulatory failure in the distal arteries. If so, it is necessary to modify the therapeutic guidelines accordingly for patients at higher risk of lower limb complications.

## 5. Summary and Future Directions

DM induces hyperglycemia and tubular growth, which enhance the amount of sodium and glucose reabsorbed by the proximal tubule, and in turn increase GFR through the physiology of TGF and tubular back pressure. Consequently, higher tubular rates of transport and oxygen consumption ensue. SGLT2i offer a myriad of direct and indirect and renoprotective effects, thus halting the progression of CKD, even in patients without DM. Particular attention was paid to transient GFR reduction, which in fact contributes to GFR preservation in the long term. Importantly, the use of mathematical modeling provided new insights that explain why renoprotective effects that arise from SGLT2 inhibition are preserved in CKD, in spite of reduced blood glucose effects. On the other hand, it was brought into question whether SGLT2i might contribute to AKI in susceptible patients, such as volume depleted or patients receiving radiocontrast agents, especially considering the hypoxic effect of SGLT2i on the outer medulla. Nevertheless, although these claims have been disproved by clinical evidence, further consideration in this regard, but also concerning euglycemic DKA, is warranted. Currently, selective SGLT1 inhibition, as well as dual inhibition of SGLT2/SGLT1 are also being explored as potential new therapeutic strategies in CKD. Furthermore, in the upcoming years, we will also have an answer as to whether SGLT2i may be valuable for patients with type 1 DM. In summary, although SGLT2i provide clinicians with an exciting new treatment option for patients with CKD, further research is needed to determine which subgroups of patients with CKD will benefit the most, and which the least, from this option.

## Figures and Tables

**Table 1:** Summary of clinical evidence concerning the role of SGLT2i in CKD.

	Study	Study Design	Study Population	SGLTi Used	Main Outcomes
CKD with DM	EMPA-REG OUTCOME trial [105]	Randomized, double-blind placebo-controlled trial	6185 patients eGFR > 30 mL/min/1.73 m^2^	Empagliflozin 10/25 mg daily	Slower progression of kidney disease and lower rates of clinically relevant renal events
	Petrykiv et al. [99]	Double-blind, placebo-controlled crossover trial	33 patients UACR > 100 mg/g RAAS blockade therapy	Dapagliflozin 10 mg daily for 6 weeks	Reduced UACR by 36.2%, SBP by 5.2 mm Hg and eGFR by 5.3 mL/min/1.73 m^2^ All effects reversible with discontinuation
	DECLARE TIMI-58 trial [35]	Randomized, double-blind placebo-controlled trial	17,160 patients eGFR ≥ 60 mL/min/1.73 m^2^ atherosclerotic CV disease or multiple risk factors	Dapagliflozin 10 mg daily	Lower risk of ESRD or renal death in dapagliflozin group Mean decrease in eGFR was larger after 6 months, equalized by 2 years, and smaller after 3 years
	EMPEROR-Reduced trial [106]	Randomized, double-blind placebo-controlled trial	3730 patients HFrEF ≤ 40%	Empagliflozin 10 mg daily	Lower annual decline in eGFR More frequent genital tract infections
	CREDENCE trial [108]	Randomized, double-blind, trial	4401 patients eGFR 30–90 mL/min/1.73 m^2^ UACR 300–5000 mg/g	Canagliflozin 100 mg daily	Lower risk of ESRD, doubling of the creatinine level or death of renal causes
CKD without DM	DIAMOND trial [112]	Randomized, double-blind, placebo-controlled crossover trial	53 adults proteinuria 500–3500 mg/24 h eGFR > 25 mL/min/1.73 m^2^ RAAS blockade therapy	Dapagliflozin 10 mg daily for 6 weeks	1.5 kg reduction in body weight No significant change in proteinuria 6.6 mL/min/1.73 m^2^ fall in eGFR, reversed after another 6 weeks No significant change in ABP
	DAPA-HF trial [114]	Double-blind, placebo-controlled, event-driven trial	4742 adults HFrEF ≤ 40% eGFR ≥ 30 mL/min/1.73 m^2^ SBP ≥ 95 mm Hg	Dapagliflozin 10 mg daily	Lower rate of decline in eGFR per year Outcomes did not differ by baseline eGFR category
	DAPA-CKD trial [115]	Randomized, double-blind placebo-controlled multricentre trial	4304 adults eGFR 25–75 mL/min/1.73 m^2^ UACR 200–5000	Dapagliflozin 10 mg daily	Lower risk of a sustained decline in eGFR Lower risk of ESRD Lower risk of death from renal or cardiovascular causes Outcomes did not differ depending on the presence of T2DM

Abbreviations: CKD: chronic kidney disease; DM: diabetes mellitus; EMPA-REG OUTCOME: Efficacy and Safety of Empagliflozin in Patients With Type 2 diabetes and Renal Impairment; UACR: urinary albumin to creatinine ratio; eGFR: estimated glomerular filtration rate; CV: cardiovascular; RAAS: renin-angiotensine-aldosterone system; CREDENCE: Canagliflozin and Renal Events in Diabetes with Established Nephropathy Clinical Evaluation; SBP: systolic blood pressure; DECLARE-TIMI: Dapagliflozin Effect on CardiovascuLAR Events; ERSD: end-stage renal disease; AKI: acute kidney injury; HFrEF: heart failure with reduced ejection fraction; DIAMOND: Dapagliflozin in Non-diabetic Patients With Proteinuria; DAPA-HF: Dapagliflozin and Prevention of Adverse Outcomes in Heart Failure; SBP: systolic blood pressure; T2DM: type 2 diabetes mellitus.

## Data Availability

Not applicable.
